# Host pre‐conditioning improves human adipose–derived stem cell transplantation in ageing rats after myocardial infarction: Role of NLRP3 inflammasome

**DOI:** 10.1111/jcmm.15403

**Published:** 2020-10-06

**Authors:** Tsung‐Ming Lee, Horng‐Jyh Harn, Tzyy‐Wen Chiou, Ming‐Hsi Chuang, Chun‐Hung Chen, Chi‐Hsuan Chuang, Po‐Cheng Lin, Shinn‐Zong Lin

**Affiliations:** ^1^ Cardiovascular Institute An Nan Hospital China Medical University Tainan Taiwan; ^2^ Department of Medicine China Medical University Taichung Taiwan; ^3^ Bioinnovation Center Tzu Chi Foundation Hualien City Taiwan; ^4^ Department of Pathology Buddhist Tzu Chi General Hospital Tzu Chi University Hualien City Taiwan; ^5^ Department of Life Science and Graduate Institute of Biotechnology National Dong Hwa University Hualien Taiwan; ^6^ Department of Technology Management Chung Hua University Hsinchu Taiwan; ^7^ Gwo Xi Stem Cell Applied Technology Hsinchu Taiwan; ^8^ Genomics Research Center Academia Sinica Taipei Taiwan; ^9^ Department of Neurosurgery Buddhist Tzu Chi General Hospital Tzu Chi University Hualien City Taiwan

**Keywords:** adipose‐derived stem cell, butylidenephthalide, myocardial fibrosis, NLRP3 inflammasome, reactive oxygen species

## Abstract

Functional decline of stem cell transplantation in ageing hosts is well documented. The mechanism for this is poorly understood, although it is known that advancing age does not provide an optimal milieu for exogenous stem cells to survive, engraft and differentiate. We showed that *n*‐butylidenephthalide improved human adipose–derived stem cell (hADSC) engraftment via attenuating the production of reactive oxygen species (ROS). It remained unclear whether pre‐treated hosts with *n*‐butylidenephthalide can rejuvenate the ageing heart and improve hADSC engraftment by regulating the ROS/NLRP3 inflammasome‐mediated cardiac fibrosis after myocardial infarction. One hour after coronary ligation, hADSCs were transplanted into the hearts of young and ageing Wistar rats that were pre‐treated with or without *n*‐butylidenephthalide for 3 days. At day 3 after infarction, myocardial infarction was associated with an increase in ROS levels and NLRP3 inflammasome activity with age. hADSC transplant effectively provided a significant decrease in ROS levels, NLRP3 inflammasome activity, IL‐1β levels and cardiac fibrosis in either young or old infarcted rats. However, the beneficial effects of hADSCs were greater in young compared with old rats in terms of NLRP3 inflammasome activity. The infarcted ageing rats pre‐conditioned by *n*‐butylidenephthalide improved engraftment and differentiation of hADSCs and additionally attenuated cardiac fibrosis compared with hADSCs alone. The anti‐inflammation effects of *n*‐butylidenephthalide were reversed by SIN‐1. In conclusions, the increased NLRP3 inflammasome activity plays the pathogenesis of ageing‐related functional hADSC decline in the ageing hosts. *n*‐butylidenephthalide‐pre‐treated ageing hosts reversibly ameliorate the harsh microenvironments, improve stem cell engraftment and attenuate cardiac fibrosis after myocardial infarction.

## 1 INTRODUCTION

Ageing patients have been shown to have dramatically worse cardiovascular outcomes compared with young patients.^1^ The advanced age of most patients who experience a myocardial infarction (MI) hinders the healing ability of the infarcted region of the heart.^1^ Previous studies have demonstrated an age‐dependent reduction in survival and greater cardiac fibrosis following MI.^1^ Importantly, the changes seen in the older hosts could be improved with therapy. Previous studies on whole muscle graft transplantation in mice have shown that the ability to regenerate muscles mainly depends on the host's age and that successful muscle engraftment was seen in young hosts, with failure of graft regeneration in old hosts.^2^ Other studies have also shown optimal regeneration in muscle grafts in young hosts.^3^ Although the intrinsic regenerative potential of ageing hosts appears to be largely intact, critical factors required for regeneration remained unclear. Therefore, further investigations into the molecular and cellular events post‐MI in older patients may improve the therapeutic outcomes in this population.

Cardiac remodelling after MI is a complex inflammatory process involving numerous signalling pathways.^4^ An exaggerated inflammatory response is believed to be responsible for the increased morbidity and mortality following MI. Inflammasomes are innate immune signalling pathways and cytosolic multiprotein complexes that are present mainly in macrophages.^5^ Nucleotide‐binding oligomerization domain‐like receptor with a Pyrin domain 3 (NLRP3) inflammasome requires two steps for activation: signal 1 for priming and signal 2 for activation. Priming requires an NF‐κB–activating stimulus, such as lipopolysaccharides binding to Toll‐like receptor 4, which induces elevated expression of NLRP3 and IL‐1β. NF‐κB is inactive in the cytosol due to interactions with its inhibitory protein, I‐κB. Once activated, NF‐κB dissociates from I‐κB, resulting in nuclear translocation of the heterodimer p50/p65 where it subsequently binds to decameric consensus sequences in target gene promoter/enhancer regions. After priming, canonical NLRP3 inflammasome activation requires reactive oxygen species (ROS) to activate NLRP3 and lead to the formation of the NLRP3 inflammasome complex.^6^ ROS scavenger treatment has been shown to block the activation of NLRP3 in response to a range of agonists.^7^ The structures of all inflammasomes are similar, and they are usually formed by an NLRP3, an effector component (caspase‐1), an adaptor component (ASC) and a substrate component (such as pro‐inflammatory cytokines pro‐IL‐1β/pro‐IL‐18). The inflammasomes respond to danger signals such as damage‐associated molecular pattern molecules (DAMPs) which are released by stressed or injured cells such as urate crystals, β‐amyloid, extracellular ATP and cell debris. DAMPs have been associated with endogenous sterile inflammation after MI,^8^ and NLRP3 inhibition via small interfering RNA has been shown to prevent cardiac cell death and the activation of inflammasomes, thereby ameliorating myocardial remodelling.^9^, ^10^ Thus, NLRP3 inflammasome inhibition might be a new strategy for sterile MI.

Recently, we showed that human adipose–derived stem cell (hADSC) transplantation is a promising new therapy to improve cardiac fibrosis after MI.^11^ Poor graft survival has a significant detrimental effect on the success of cell transplantation. Over 99% of mesenchymal stem cells injected into the left ventricular (LV) myocardium of mice die within four days after injection.^12^ An essential challenge would be to determine the signals controlling reduced stem cell survival. Injured myocardium represents harsh environment for the transplanted cells including inflammatory cytokines, acidosis, increased calcium and oxidative stress, hypoxia, or unbalanced supply of nutrition. IL‐1β has been shown to play a role in acute inflammation and graft death after direct intramuscular cell transplantation to the heart.^13^ Blockade of IL‐1β by neutralizing antibody improved stem cell survival and resulted in an increase in the total number of donor‐derived cells.^13^ Moreover, in mice lacking the IL‐1 receptor, markedly decreased adverse remodelling of infarcted hearts has been observed, possibly through a reduction in collagen deposition caused by the lack of IL‐1 signalling.^14^ Therefore, targeting IL‐1 as a way to modulate pathological heart remodelling may be clinically relevant.^15^

*Angelica sinensis* is a well‐known traditional Chinese herbal medicine that has been shown to have a broad spectrum of biological activities, including anti‐inflammatory properties and the ability to regulate immunity in hepatic fibrosis and cardiovascular diseases.^16^ Butylidenephthalide (BP) is the major component of *Angelica sinensis*.^17^ Very recently, we have shown that BP can attenuate superoxide production after MI.^11^ Naoxintong, an extract from 16 various kinds of Chinese traditional herbal Medicines including *Angelica sinensis,* has been shown to provide cardioprotective effect by inhibiting the NLRP3 inflammasome activity.^18^ However, whether BP alone inhibited the NLRP3 inflammasome activity remained unknown. Previous studies have shown that ageing repair can be modulated by regulation of many signalling pathways including ROS and NLRP3 inflammasome activity.^19^ Donor and recipient ageing has been shown to impair cell engraftment^20^; however, the host environment was more important in determining the regenerative efficacy than was the age of the donor.^20^ The regenerative potential of ageing stem cells can be restored by exposure to serum from young hosts, implying the importance of the host environment in regulating regeneration.^20^ Patients who suffer from MI and heart failure are generally older. Thus, it is important to delineate the role of BP in rejuvenating ageing hosts as defined by morphological and functional characteristics and expression of molecular markers. In this study, we assessed (a) whether there were differential age‐related responses of NLRP3 inflammasomes to MI, (b) whether there were different responses between young and ageing infarcted hosts in hADSC administration, and (c) whether pre‐treatment with BP in ageing hosts can improve the age‐related fibrosis by regulating NLRP3 inflammasomes in a rat MI model.

## 2 METHODS

The cell culture and animal experiment were approved and conducted in accordance with local institutional guidelines for the care and use of laboratory animals at the China Medical University (CMU‐2018‐042) and conformed with the *Guide for the Care and Use of Laboratory Animals* published by the US National Institutes of Health (NIH Publication No. 85‐23, revised 1996).

### 2.1 Isolation of hADSCs

hADSCs (StemPro human ADSC kit; Invitrogen) were generously provided by Gwo Xi Stem Cell Applied Technology as previously described.^11^ The hADSCs were cultured in growth media (low glucose DMEM (Invitrogen), 10% foetal bovine serum (Serana) and 1% penicillin/streptomycin (HyClone)) and then subcultured using TrypLE Express (Invitrogen) for subsequent passages. Passaged hADSCs were defined as being passage 1, with passages 3‐5 being used in this study. These homogeneous hADSCs did not contain haematopoietic lineages or endothelial cells. In addition, they expressed the mesenchymal stem cell marker CD90 but not haematopoietic markers CD31 or CD45, and they were confirmed to be >95% CD90^+^ and <2% CD31^+^/CD45^+^.

### 2.2 Characterization of hADSC surface phenotype

The cells (1 × 10^5^ per sample) were treated with the following specific anti‐human antibodies: anti‐isotype IgG1‐PE, anti‐CD19‐PE, anti‐CD34‐PE, anti‐CD73‐PE, anti‐CD105‐PE, anti‐isotype IgG1‐FITC, anti‐CD45‐FITC, anti‐CD90‐FITC, anti‐isotype IgG2a‐PE, anti‐CD14‐PE, anti‐HLA‐DR‐PE (BD Biosciences, San Jose, CA, USA). Mouse IgG was used as a negative control condition. For a detailed information, please refer to the Supplementary material online.

### 2.3 Animals

#### 2.3.1 Experiment 1

Myocardial infarction was induced by ligation of the anterior descending artery in young (2‐month‐old) and old (24‐month‐old) male Wistar as described previously.^11^ To avoid the possibility that the measures were due to senescent decompensation, not ageing compensation, we did not use very old rats. For surgery, haemodynamic measurements and killing, the rats were intraperitoneally anaesthetized with Zoletil (20 mg/kg bodyweight) and xylazine (9 mg/kg). Anaesthesia was tested by reflexes of the hind feet before and during the procedures, monitoring the respiratory patterns, and responses to manipulations during the procedures. The animals were ventilated with 95% O_2_ and 5% CO_2_ using a ventilator (Harvard Apparatus 486).

One hour after ligation, rats were assigned into groups: young rats (young), young rats receiving hADSC transplantation (young/ADSC), old rats (old), old rats receiving hADSC transplantation (old/ADSC), and old rats receiving hADSC transplantation and BP pre‐treatment (old (BP)/ADSC). For cell transplantation, the hADSCs were detached from the plate, suspended in 30 μL of PBS (1 × 10^6^ cells) and transplanted at three injection sites into viable myocardium bordering the infarction using a syringe with a 30‐gauge needle. BP (Alfa Aesar) was dissolved in dimethylsulphoxide (Sigma). The oral dose of BP (150 mg/kg/d) was according to previous studies,^21^ starting 3 days prior to and ending 3 days after MI. The heart was excised at days 3 or 28 after MI as early and late stages of MI.

#### 2.3.2 Experiment 2

Given both hADSC and BP provided anti‐inflammation effect, we performed additional experiments to dissect the role of BP in regulating host microenvironment without hADSC implantation. To confirm the importance of the ROS signalling in BP‐induced NLRP3 inflammasomes, we employed 3‐morpholinosydnonimine (SIN‐1, a peroxynitrite generator). Immediately after induction of MI by coronary ligation in old male Wistar adult rats (24 months old), infarcted rats were randomized to vehicle, pre‐treatment with BP, or a combination of pre‐treatment with BP and SIN‐1. BP (150 mg/kg/d) was treated 3 days prior to coronary ligation. The dose of SIN‐1 (0.1 mg/kg, single oral dose immediately after coronary ligation) has been shown to be effective in biological activity.^22^ The heart was excised one hour after MI. At the end of the study, the ROS levels were measured in all of the hearts (n = 5 in each group) and Western analysis of NF‐κB, NLRP3 and IL‐1β proteins at the border zone (<2 mm outside the infarct).

### 2.4 Echocardiogram

Echocardiography was performed at baseline before and then again at 3 and 28 days after surgery. For a detailed information, please refer to the Supplementary material online.

### 2.5 Haemodynamics and infarct size measurements

At the end of the study, the size of the infarcts and haemodynamic parameters was measured (online Supplementary material). Only rats with clinically important MIs (>30%) were selected for analysis.

### 2.6 Western blot analysis of p65 NF‐κB, NLRP3 and IL‐1β

Samples were obtained from the border zone either at day 3 in Experiment 1 or 1 hour in Experiment 2 after MI. Antibodies to p65 NF‐κB (Santa Cruz Biotechnology), NLRP3 (Santa‐Cruz Biotechnology, sc‐34408), cleaved IL‐1β (Cell Signaling Technology) and β‐actin (Santa Cruz Biotechnology) were used. Western blotting procedures were described previously.^11^ Experiments were replicated three times and results expressed as the mean value.

### 2.7 Quantitative PCR (qPCR) of human Alu, p65 NF‐κB, NLRP3 and IL‐1β

qPCR was performed from samples obtained from the border zone at day 3 as previously described.^11^ The gene expression patterns for human *Alu, p65 NF‐κB, NLRP3 and IL‐1β*were assessed. The PCR primer sequences are shown in the online data supplement.

### 2.8 Immunohistochemical analysis of human mitochondria and sarcomeric α‐actinin

Immunohistochemistry staining for human mitochondria antibody, sarcomeric α‐actinin antibody and 4′,6‐diamidino‐2‐phenylindole dihydrochloride (DAPI) was used to identify and transdifferentiate the transplanted cells at the border zone on day 28. For a detailed information, please refer to the Supplementary material online.

### 2.9 In situ detection of superoxide anion

To evaluate the production of myocardial intracellular superoxide using in situ dihydroethidium (DHE, 1 µmol/L, Invitrogen Molecular Probes) fluorescence, 5‐µm paraffin‐embedded tissue sections were incubated with DHE in PBS (10 mmol/L) in a dark, humidified container at room temperature for 30 minutes. Superoxide radical generation in the tissues was assessed by the presence of a red fluorescent signal, and image density was presented as arbitrary units/mm^2^ field.^23^ DHE fluorescence was digitally recorded using an Olympus microscope. To minimize interference by non‐specific DHE oxidation products, a red fluorescence was detected with excitation line 405 nm.^24^ The fluorescence intensity of nuclei from each section was measured and was corrected for background fluorescence in nonnuclear regions with Image‐Pro Plus software (Media Cybernetics). Four sections per rat were studied.

### 2.10 Morphometry of cardiac fibrosis

Picrosirius staining and aniline blue staining were used to stain cardiac fibrosis from the remote zone (>2 mm within the infarct) at day 28 after MI. For a detailed information, please refer to the Supplementary material online.

### 2.11 Laboratory measurements

Serum IL‐1β levels at day 3 after MI were assessed using ELISA kits (R&D Systems, Minneapolis, MN, USA) and expressed as pg/mL.

Collagen histology was confirmed by hydroxyproline assay using a method adapted from that reported by Stegemann and Stalder.^25^ Remote zone samples were immediately frozen in liquid nitrogen and stored at −80°C until the hydroxyproline content per weight of tissue was measured.

Myocardial superoxide production at the remote zone was measured using lucigenin (5 μmol/L bis‐N‐methylacridinium nitrate, Sigma) enhanced chemiluminescence as previously described.^23^ The chemiluminescence in the sample was continuously measured for a total of 300 seconds. The specific chemiluminescence signal was calculated after subtracting background activity and was expressed as counts/min/mg weight (cpm/mg).

### 2.12 Statistical analysis

The results were presented as mean ± SD. Statistical analysis was performed using SPSS version 19 (SPSS). Differences among the groups of rats were tested using ANOVA. If an *F* value was significant for the main effect or the interaction, between‐group differences were tested using Tukey's multiple range post hoc test. The significant level was assumed at a *P* value of < 0.05.

## 3 RESULTS

### 3.1 Part 1: acute stage (day 3)

Heart tissue was harvested at day 3 after MI. Differences in mortality rate (2/7 (29%), 2/7 (29%), 1/6 (17%), 3/8 (38%) and 2/7 (29%) in young, young/ADSC, old, old/ADSC and old (BP)/ADSC, respectively; *P* = NS) and infarct size (33 ± 2%, 34 ± 2%, 35 ± 3%,, 34 ± 3% and 34 ± 2%, in young, young/ADSC, old, old/ADSC and old (BP)/ADSC, respectively; *P* = NS) among the infarcted groups were not found at the acute stage of MI.

#### 3.1.1 Effect of ageing hosts and BP‐pre‐conditioned hosts on hADSC engraftment

**FIGURE 1:**
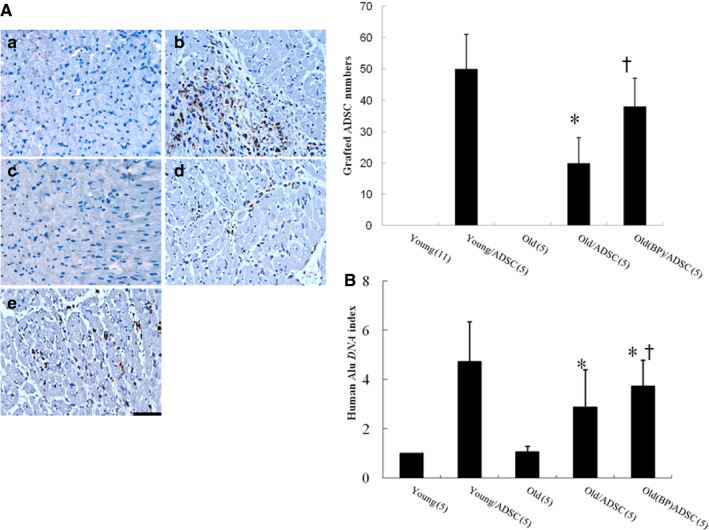
A, Identification of the transplanted hADSCs and their cardiac phenotype from the border zone 3 d after infarction. The grafted cells were identified, and their human nature was established by positive immunostaining with anti‐human mitochondrial antibodies (brown). Human stem cells were observed at implanted area of transplantation groups but not the vehicle. More grafted cells were identified in the infarcted group pre‐treated with BP compared with hADSCs alone. A, young; B, young/ADSC; C, old; D, old/ADSC; E, old(BP)/ADSC. B, Engraftment of hADSCs. qPCR for human *Alu* was performed. Alu‐DNA index (amount of *Alu*‐amplified DNA in hADSC recipient rats relative to that detected in young infarcted rats). The number of animals in each group is indicated in parentheses. *P < .05 compared with young/ADSC; ^†^ *P* < .05 compared with the infarcted group treated with old/ADSC

To quantify the total number of transplanted hADSCs (surviving and proliferating), the human origin of these transplanted cells was confirmed by staining with anti‐human mitochondria antibodies and the human *Alu* gene 3 days after transplantation. Human stem cells were observed at the implanted areas in the transplantation group but not the vehicle group (Figure 1). In the transplanted hearts, a small number of specific mitochondria‐positive human cells were retrieved in clusters, mainly at the border zone (Figure 1A). Compared with young vehicle, ageing rats showed significantly lower infiltration of the mitochondria‐positive cells. The reduced infiltration of the mitochondria‐positive cells was improved after administering BP. The number of mitochondria‐positive cells in old hosts pre‐treated with BP was 38 ± 9, significantly higher than that without (20 ± 8, *P* < .05). These data suggest that BP‐primed hosts increase the survival of hADSCs in post‐infarct old hearts.

qPCR analysis of human *Alu* sampled from the border zone was performed on day 3 after infarction. The results revealed the presence of human *Alu* DNA sequences in the myocardium of all hADSC recipients, albeit at low levels (Figure 1B). The fractions of human *Alu* genomic DNA relative to total myocardium DNA were significantly higher in the BP‐pre‐treated group compared to the hADSCs alone.

#### 3.1.2 Effect of ageing hosts and BP‐pre‐conditioned hosts on ROS, NF‐κB, NLRP3 inflammasome and IL‐1β after hADSC transplantation

**FIGURE 2:**
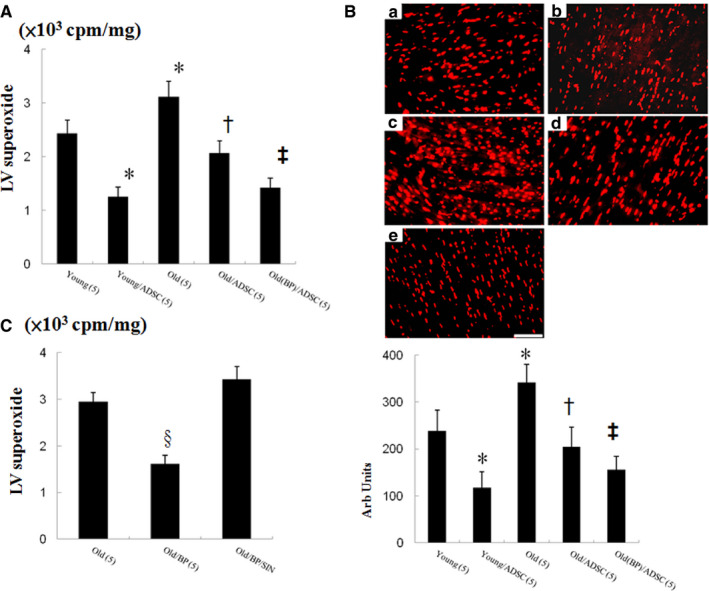
Experiment 1. A, Myocardial superoxide measured by lucigenin‐amplified chemiluminescence. B, DHE staining from the border zone 3 d after infarction (n = 5 each group). Experiment 2. C, Myocardial superoxide measured by lucigenin‐amplified chemiluminescence. The number of animals in each group is indicated in parentheses. *P < .05 compared with young; ^†^ *P* < .05 compared with the infarcted groups treated with young/ADSC and old; ^‡^ *P* < .05 compared with old/ADSC. ^§^ *P* < .05 compared with the infarcted groups treated with old and old/BP/SIN‐1

Superoxide production was assessed by lucigenin‐enhanced chemiluminescence and DHE staining. Compared with young vehicle, the magnitude of increased ROS level was higher in the ageing infarcted hearts (Figure 2A). Thus, although ageing hearts have similar trends in responses to injury, the responses in ageing hearts were overreactive. Besides, the increased ROS levels were reversed after adding hADSC*‐*treated infarcted group compared with vehicle in ageing rats. BP pre‐treatment further decreased ROS levels to 31 ± 11% of old/ADSC (*P* < .05). DHE staining mirrored the results of lucigenin‐enhanced chemiluminescence (Figure 2B). The experiment 2 showed that the attenuated effect of BP on superoxide production was abolished after adding SIN‐1 (Figure 2C).

**FIGURE 3:**
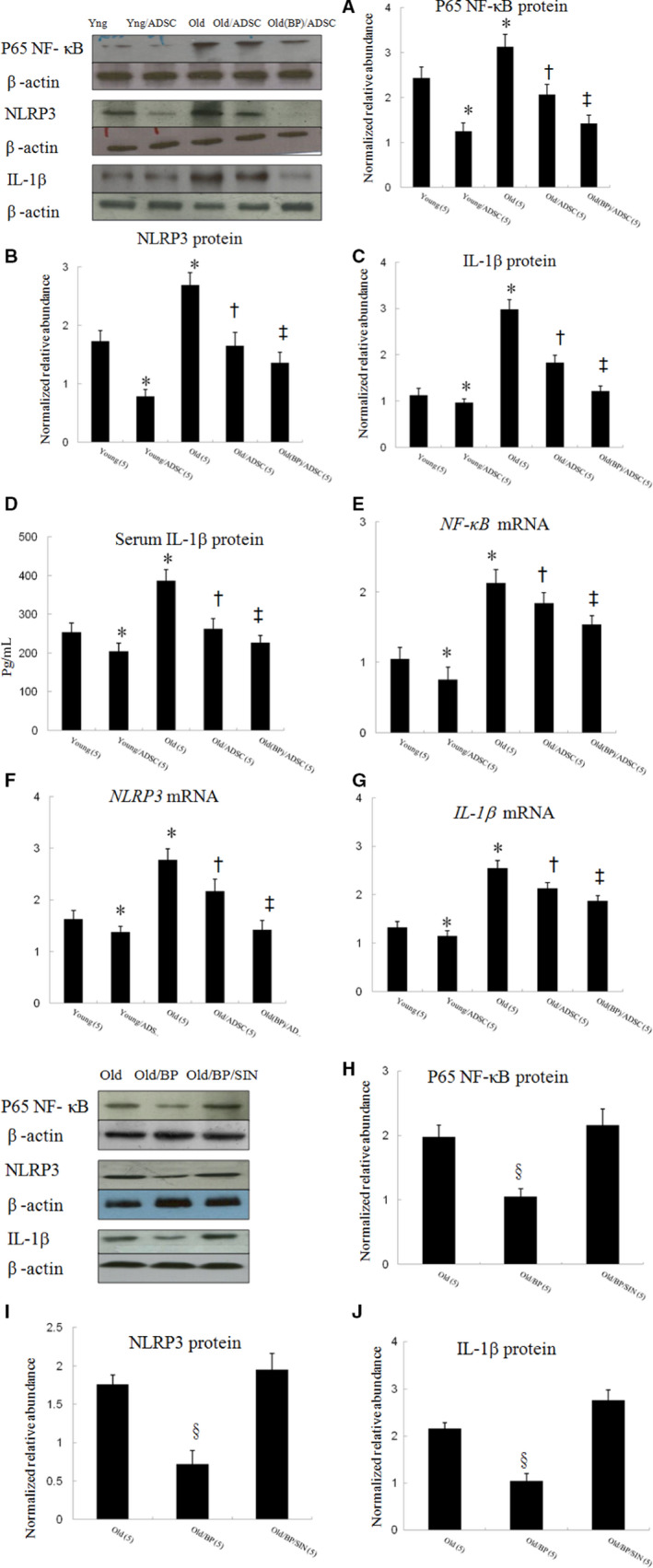
Experiment 1. The changes in NLRP3 inflammasome at day 3 from the border zone. The protein levels of NF‐κB (A), NLRP3 (B) and IL‐1β (C) assessed by Western blot were significantly increased in old than young rats after infarction. Relative abundance was obtained against that of β‐actin. To further confirm serum IL‐1β levels, ELISA was performed (D). The mRNA changes in *NF‐κB* (E), *NLRP3* (F) and *IL‐1β* (G) were similar to those of proteins. Results are mean ± SD of 3 independent experiments (n = 5 each group). Experiment 2. Pre‐treated hosts with BP provided reduction of these cytokines, which can be reversed by adding SIN‐1 (H‐J). The number of animals in each group is indicated in parentheses. *P < .05 compared with young; ^†^ *P* < .05 compared with the infarcted groups treated with young/ADSC and old; ^‡^ *P* < .05 compared with old/ADSC; ^§^ *P* < .05 compared with the infarcted groups treated with old and old/BP/SIN‐1

Then, we evaluated the protein and mRNA expression of NLRP3 inflammasome components by Western blot and qPCR. Compared with young vehicle‐treated rats, the protein levels of p65 NF‐κB (Figure 3A), NLRP3 (Figure 3B) and IL‐1β (Figure 3C) were significantly increased compared with those in the ageing infarcted hearts, indicating that the NLRP3 inflammasome was significantly overactivated in the ageing infarcted hearts. hADSC transplant effectively provided a significant decrease in p65 NF‐κB, NLRP3 inflammasome activity, myocardial IL‐1β levels in either young or old infarcted rats. The beneficial effects of hADSCs were greater in young (55 ± 8%) compared with old (39 ± 7%, *P* < .05) rats in terms of inhibiting NLRP3 inflammasome activity. BP primed hosts resulted in a decrease in p65 NF‐κB, NLRP3 and IL‐1β levels compared with that in hADSCs alone (Figure 3A‐C, all *P* < .05). Serum IL‐1β levels were verified by ELISA assay (Figure 3D), which was consistent with the Western blotting results. *p65 NF‐κB, NLRP3 and IL‐1β*mRNA were significantly increased in old infarcted rats compared to young infarcted rats (Figure 3E‐G). hADSC transplantation showed significant reduction in *p65 NF‐κB, NLRP3 and IL‐1β*mRNA in either young or ageing hosts. Besides, pre‐conditioning with BP provided additional reduction in these inflammation‐related mRNAs.

The protein levels of p65 NF‐κB, NLRP3 and IL‐1β can be reduced after administering BP as shown in Experiment 2 (Figure 3H‐J), which can be reversed after adding SIN‐1.

### 3.2 Part 2: chronic stage (day 28)

No tumours were seen during necropsy at the end of the study in any of the animals. No significant differences in mortality among the infarcted groups were found throughout the study (Table 1). In addition, there were no significant differences in relative heart weights normalized by bodyweight at the end of the experimental period among groups (data not shown). Four weeks after infarction, the infarcted area of the LV was very thin and had been totally replaced by fully differentiated scar tissue. The weights of the LV including the septum remained basically constant at 4 weeks among the infarcted groups. Compared with vehicle, the lung weight/bodyweight ratio, an index of lung oedema, was significantly lower in the groups with hADSC. The values of +dp/d*t* and −dp/d*t* were significantly higher in the infarcted groups with hADSCs compared with those without. The values of the right ventricular weight/bodyweight ratio, the lung weight/bodyweight ratio +dp/d*t* and −dp/d*t* were significantly improved after administering BP compared with those old infarcted rats treated with hADSCs alone. LV end‐systolic pressure, LV end‐diastolic pressure and infarct size did not differ among the infarcted groups.

**TABLE 1:** Cardiac morphometry and haemodynamics at the end of study

Parameters	Young	Young/ADSC	Old	Old/ADSC	Old (BP)/ADSC
No. of rats	11	12	11	10	10
Mortality, n (%)	5 (31%)	4 (25%)	3 (21%)	3 (23%)	4 (29%)
Bodyweight, g	288 ± 12	295 ± 18	604 ± 24*, †	582 ± 18*, †	597 ± 23*, †
HR, bpm	405 ± 14	402 ± 12	387 ± 12	392 ± 14	405 ± 18
LVESP, mm Hg	95 ± 5	97 ± 5	94 ± 6	96 ± 7	96 ± 5
LVEDP, mm Hg	18 ± 5	15 ± 3	19 ± 3	18 ± 5	16 ± 4
LVW/BW, mg/g	2.64 ± 0.29	2.78 ± 0.24	2.78 ± 0.22	2.62 ± 0.19	2.79 ± 0.26
RVW/BW, mg/g	0.94 ± 0.26	0.61 ± 0.24*	1.25 ± 0.19*, †	0.89 ± 0.18^†^, ^‡^	0.70 ± 0.22*, §
LungW/BW, mg/g	7.54 ± 0.54	5.17 ± 0.49*	8.73 ± 0.38*, †	5.82 ± 0.40^†^, ^‡^	4.92 ± 0.44*, §
+dp/d*t*, mm Hg/s	2341 ± 234	3183 ± 255*	2012 ± 251*, †	2873 ± 254^†^, ^‡^	3282 ± 265*, §
−dp/d*t*, mm Hg/s	2115 ± 258	2872 ± 262*	1663 ± 223*, †	2408 ± 283^†^, ^‡^	2748 ± 238*, §
Infarct size, %	40 ± 2	41 ± 3	38 ± 3	40 ± 3	41 ± 2

Note

Values are mean ± SD.

Abbreviations: BP, n*‐*butylidenephthalide; BW, bodyweight; HR, heart rate; LungW, lung weight; LVEDP, left ventricular end‐diastolic pressure; LVESP, left ventricular end‐systolic pressure; LVW, left ventricular weight; RVW, right ventricular weight.

* *P* < .05, compared with young.

^†^ *P* < .05, compared with the infarcted group treated with young/ADSC.

^‡^ *P* < .05 compared with old.

^§^ *P* < .05 compared with old/ADSC.

#### 3.2.1 Effect of ageing hosts and BP‐pre‐conditioned hosts on the long‐term transdifferentiation

**FIGURE 4:**
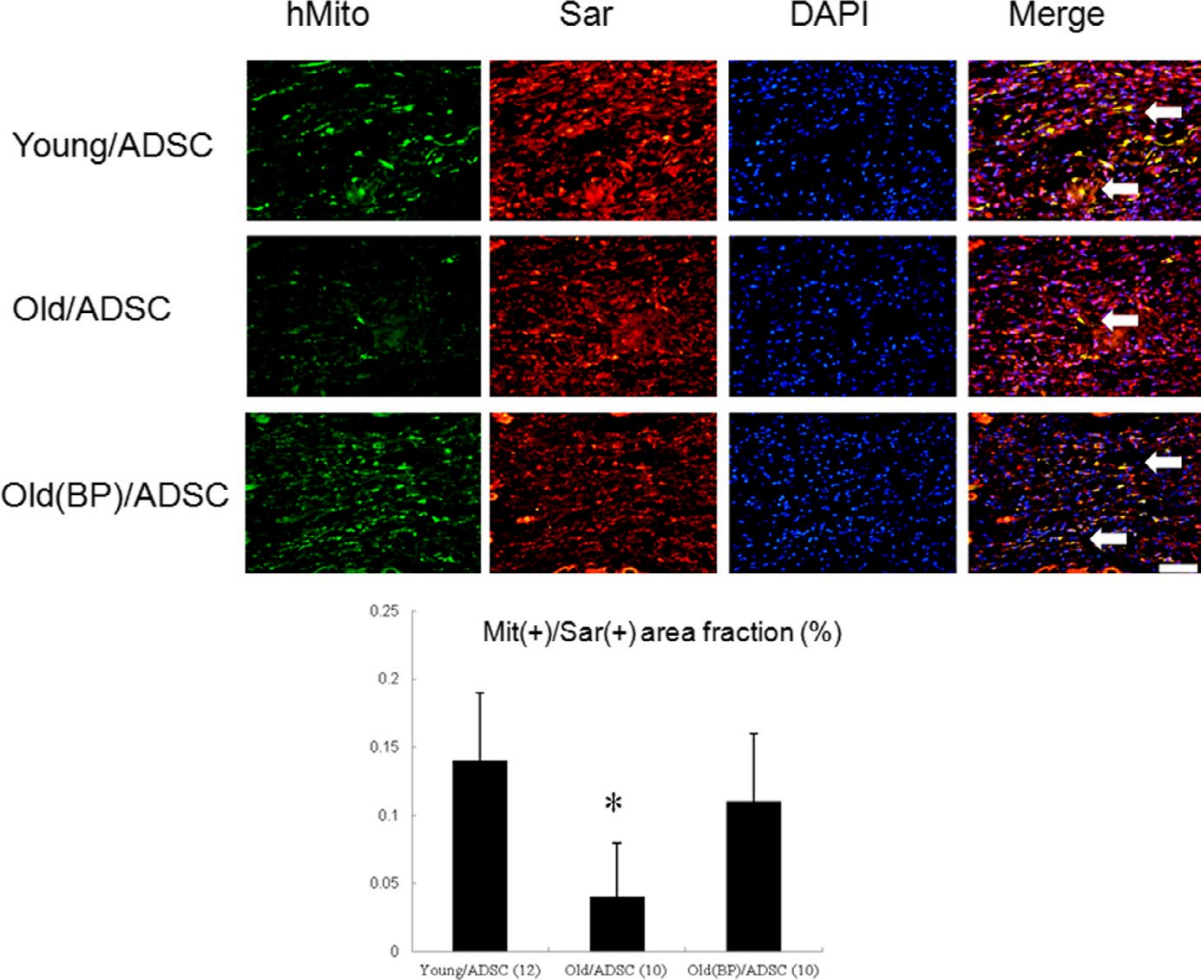
Immunofluorescent staining for human mitochondria (green) and sarcomeric α‐actinin (red), and DAPI (blue). Identification of cardiac phenotype of the transplanted hADSCs 28 d after infarction. Cardiomyocyte phenotype of transplanted hADSCs was confirmed by coexpression of human mitochondria (green) and sarcomeric α‐actinin (cardiomyocyte phenotype, red). Visualization of nuclei with DAPI stain. In the merged image, coexpression of human mitochondria and sarcomeric α‐actinin showed co‐localization of antigen expression as indicated by yellow fluorescence. The number of animals in each group is indicated in parentheses. *P < .05 compared with infarcted rats treated with young/ADSC and old(BP)ADSC

To assess transdifferentiation of hADSC‐derived ventricular cardiomyocytes, cardiomyocyte phenotype was confirmed by the coexpression of human mitochondria and sarcomeric *α*‐actinin antibodies. In some engrafted cells, co‐localization of human cell marker and cardiomyocyte‐specific marker was observed (Figure 4). Compared with old/ADSCs alone, BP primed hosts showed a higher transdifferentiation rate (0.11 ± 0.05% vs. 0.04 ± 0.04% in hADSCs alone, *P* < .001).

#### 3.2.2 Effects of ageing hosts and BP‐pre‐conditioned hosts on cardiac function

**FIGURE 5:**
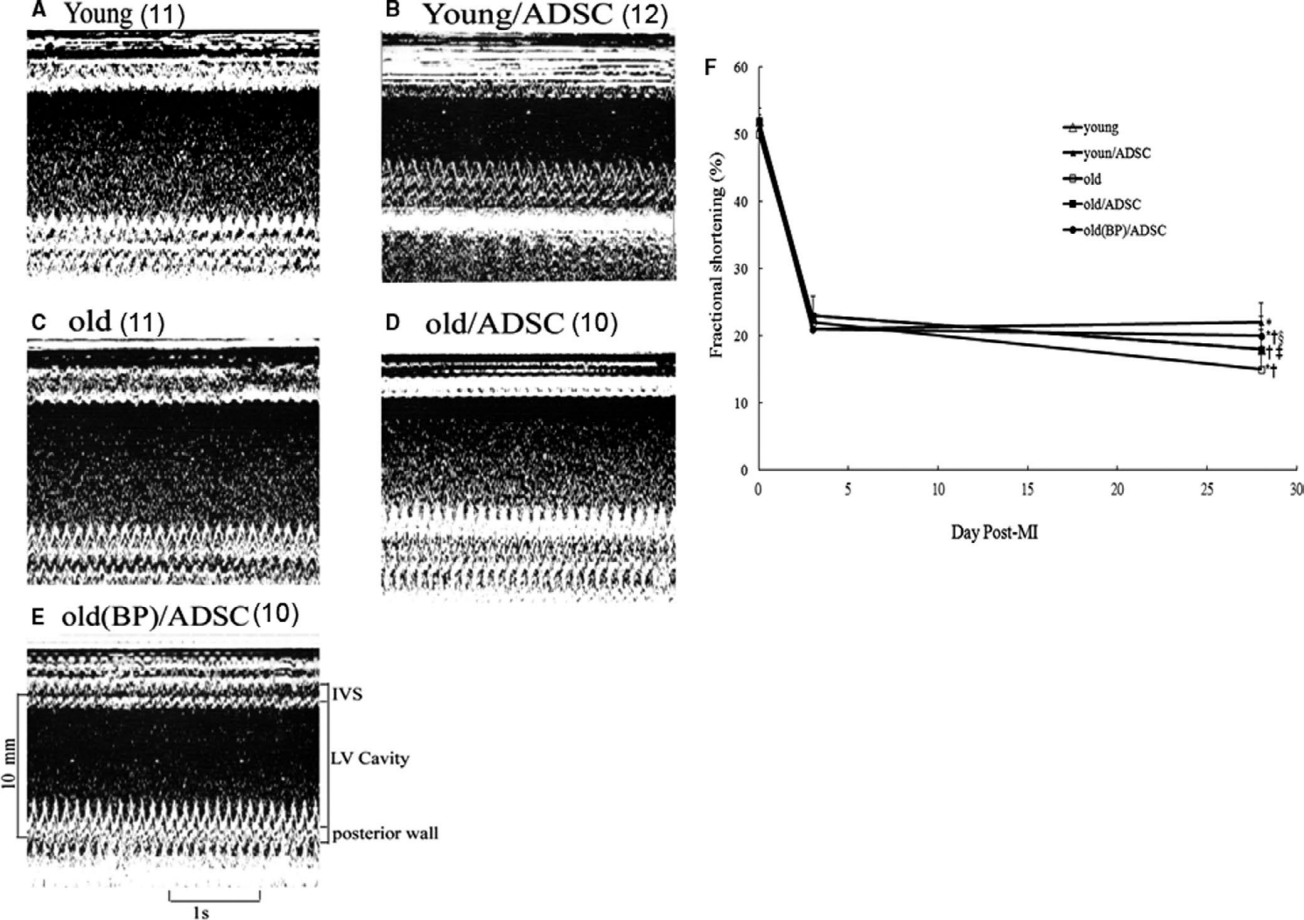
Serial echocardiographic analysis of heart function. A‐E, M‐mode echocardiographic images obtained at 28 d after MI. A severe hypokinesia to akinesia in the interventricular (IVS) wall motion was detected. F, Fractional shortening as determined from 2D images at baseline before and then again at 3 and 28 d after surgery. The number of animals in each group is indicated in parentheses. *P < .05 compared with young at the same time point; ^†^ *P* < .05 compared with the infarcted group treated with young/ADSC at the same time point; ^‡^ *P* < .05 compared with old at the same time point; ^§^ *P* < .05 compared with old/ADSC at the same time point

To determine whether BP‐pre‐treated hosts generated an enhanced therapeutic effect of stem cells, we evaluated the effects of hADSC transplantation on cardiac function in post‐MI rat hearts (Table 2, Figure 5). LV fractional fraction was significantly decreased in the old infarcted rats compared with young infarcted rats. Compared with young infarcted rats, ageing rats had significant increase in LV end‐systolic dimension and LV end‐diastolic dimension. Fractional fraction was significantly improved in the hADSC‐treated groups with a significant improvement of fractional fraction in the young group compared with the old group. Fractional fraction was further improved in the old rats pre‐treated with BP compared with those in hADSCs alone.

**TABLE 2:** Echocardiographic findings at the end of study

Parameters	Young	Young/ADSC	Old	Old/ADSC	Old (BP)/ADSC
No. of rats	11	12	11	10	10
Interventricular septum (mm)	0.5 ± 0.2	0.6 ± 0.2	0.6 ± 0.2	0.6 ± 0.2	0.6 ± 0.2
LVEDD (mm)	9.3 ± 0.2	7.8 ± 0.2*	9.7 ± 0.2*, †	9.1 ± 0.3^†^, ^‡^	9.0 ± 0.2*, †
LVESD (mm)	7.6 ± 0.2	6.1 ± 0.2*	8.2 ± 0.3*, †	7.5 ± 0.2^†^, ^‡^	7.2 ± 0.2*, †
LV posterior wall (mm)	1.7 ± 0.2	1.7 ± 0.2	1.7 ± 0.2	1.8 ± 0.1	1.8 ± 0.2
Fractional shortening (%)	18 ± 3	22 ± 3*	15 ± 4*, †	18 ± 3^†^, ^‡^	20 ± 3*, †, §

Note

Values are mean ± SD. Abbreviations as in Table 1.

Abbreviations: LVEDD, left ventricular end‐diastolic dimension; LVEF, left ventricular ejection fraction; LVESD, left ventricular end‐systolic dimension.

* *P* < .05 compared with young.

^†^ *P* < .05 compared with the infarcted group treated with young/ADSC.

^‡^ *P* < .05 compared with old.

^§^ *P* < .05 compared with old/ADSC.

#### 3.2.3 Effects of ageing hosts and BP‐pre‐conditioned hosts on remote myocardial fibrosis

**FIGURE 6:**
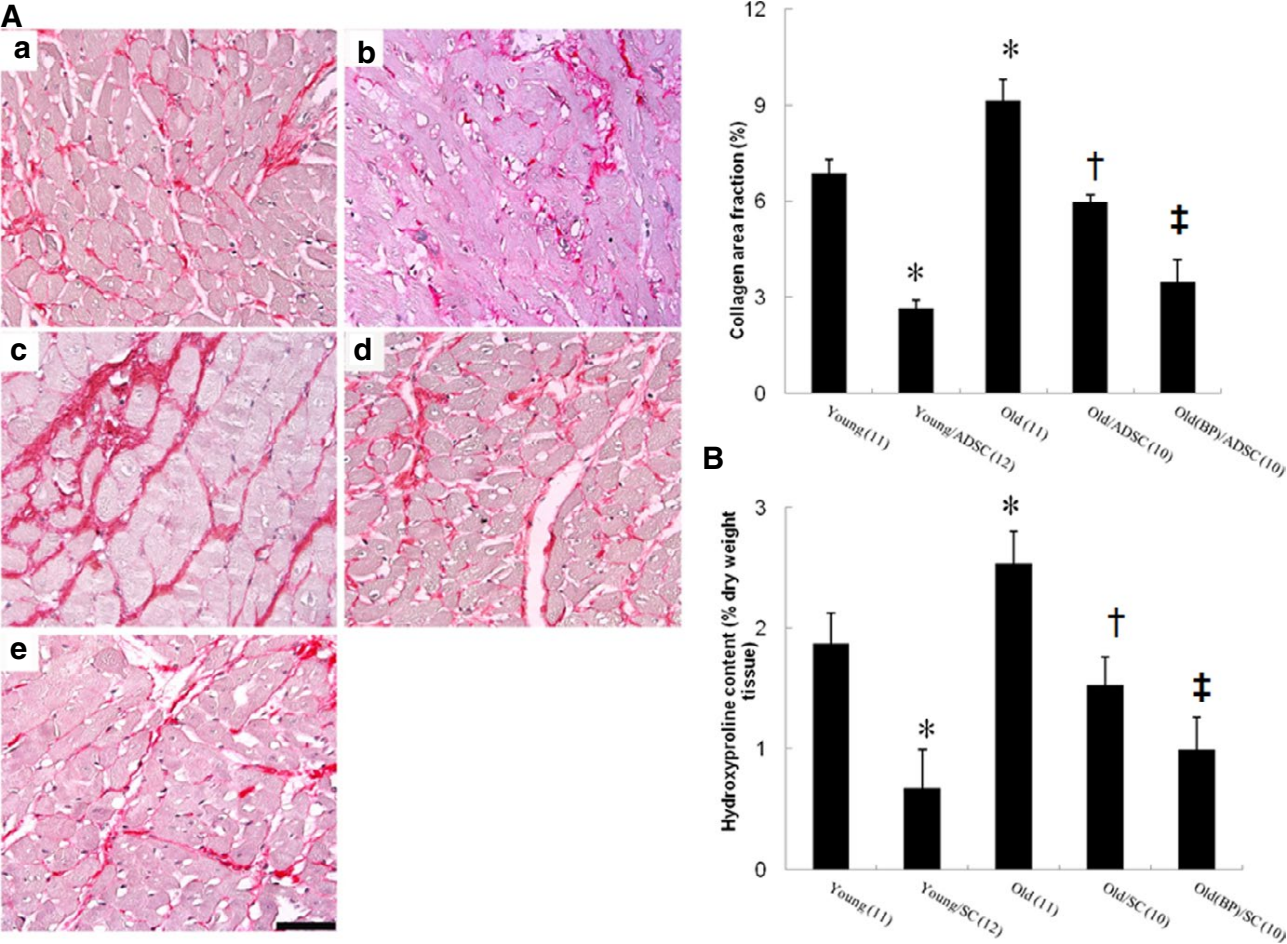
Cardiac fibrosis at the remote zone at day 28 after infarction. A, Representative sections from the remote zone with Sirius Red staining (red, magnification 400×). The line length corresponds to 50 μm. The values are mean ± SD of 5 animals from each group. B, Hydroxyproline content was also shown to measure quantitative amount of fibrosis. The number of animals in each group is indicated in parentheses. *P < .05 compared with young; ^†^ *P* < .05 compared with the infarcted groups treated with young/ADSC and old; ^‡^ *P* < .05 compared with old/ADSC

Sirius Red staining showed LV fibrosis at the remote zones of the tissue sections (Figure 6A). Old infarcted rats treated with vehicle had significantly larger areas of intense focal fibrosis compared with young infarcted rats (9.15 ± 0.66% vs. 6.87 ± 0.43%, *P* < .05). Compared with hADSCs alone in old infarcted rats, treatment with BP‐pre‐treated rats further attenuated fibrosis. Hydroxyproline analysis also showed a marked increase in collagen content in the old rats compared to the young rats after MI, and this was significantly attenuated by either hADSC or BP pre‐treatment (Figure 6B).

## 4 DISCUSSION

We demonstrated for the first time that excessive NLRP3 inflammasome activities may be responsible for ageing‐related decline in stem cell engraftment, which can be reversed to levels in young animals by pharmacological pre‐treatment with BP. Our results were consistent with the notion that the ageing heart is more refractory to regenerative therapy. Increased ROS and NLRP3 inflammasome activities in ageing host environment may have deleterious functions on transplanted hADSCs. Inhibition of NLRP3 inflammasome rejuvenated the behaviour of hADSCs, reinforcing the idea that age‐related inflammatory responses become counterproductive for cardiac fibrosis.

**FIGURE 7:**
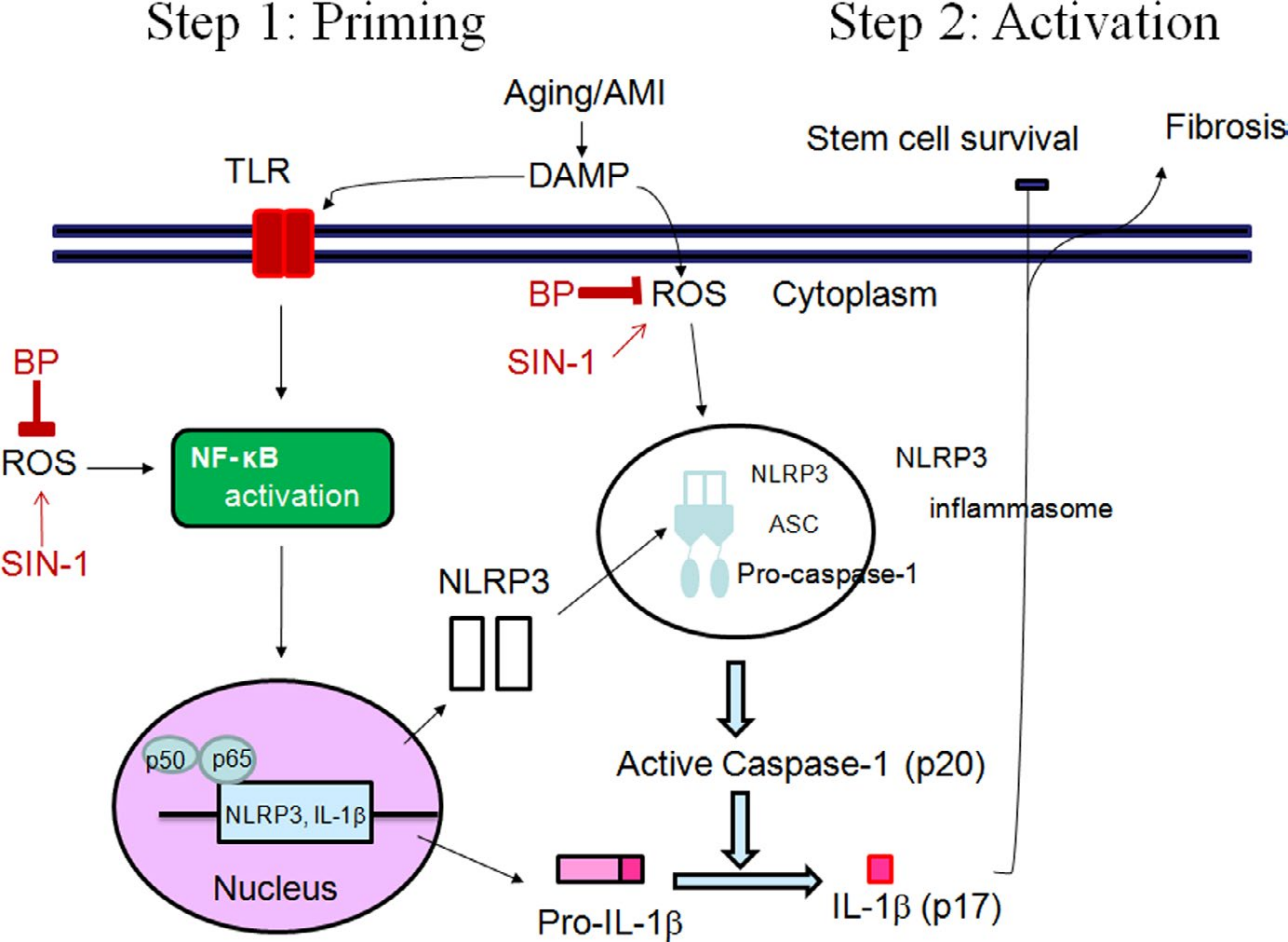
Schematic representation illustrates the involvements of BP‐mediated NLRP3 inflammasomes in cardiac fibrosis in postinfarcted rats. Activation of NLRP3 inflammasome, composed of NLRP3, ASC and pro‐caspase‐1, is tightly regulated by two‐step signals. The first “priming” signal, such as MI‐mediated DAMP, enhances the expression of inflammasome components and target proteins via activation of transcription factor NF‐κB. The second “activation” signal activates NLRP3 which recruits the ASC scaffolding protein and procaspase‐1 allowing for homodimerization and autocatalytic activation of caspase‐1. Active caspase‐1 cleaves pro–IL‐1β into the active isoform. BP inhibits ROS production, which in turn suppresses two‐step signals of NLRP3 inflammasome activation. Inhibition of these signalling pathways by their respective inhibitors is indicated by the vertical lines. TLR, Toll‐like receptor; DAMPs, danger‐associated molecular patterns

Our identification of the ROS/NLRP3 inflammasome pathway as a necessary regulator of hADSC survival provides new insights into cardiac collagen deposition and fibrosis formation after MI. BP pre‐treatment in hosts has also been shown to inhibit MI‐induced cardiac fibrosis through a ROS/NLRP3‐dependent mechanism, suggesting that strategies to inhibit the ROS/NLRP3 pathway may lead to novel and efficacious treatments for MI. Our conclusions are supported by three lines of evidence (Figure 7).

### 4.1 Effect of ageing on cardiac NLRP3 inflammasome changes in infarcted rats

Our results showed that more structural and functional changes were observed in ageing rats compared with young rats assessed by inflammation activity at day 3 and cardiac fibrosis at day 28 after MI. We evaluate the ROS/NLRP3 signalling pathway in the ageing rat heart and to explore the potential therapeutic role of this signalling pathway in cardiac rejuvenation and tissue regeneration after myocardial injury. Mitochondrial overproduction of ROS has been shown to contribute to cellular senescence.^26^ ROS overproduction ultimately leads to formation of the highly reactive product superoxide anions, whose accumulation and diffusion foster inflammation. Even though various hypotheses have been proposed to explain the ageing process, ROS are still regarded to be the main cause of ageing.^27^ Previous studies have shown that an increase in ROS generation is a key factor in regulating the secretion of NLRP3 inflammasome‐activated IL‐1β in the innate immune system.^28^ We found that NLRP3 levels after MI in old rats were significantly increased than those in young rats. Thus, interventions that reduce NLRP3 levels in the old rat heart may confer beneficial effects. Previous studies have shown that ablation of NLRP3 inflammasome protected mice from age‐related increases in systemic inflammation.^29^

### 4.2 Effect of hADSC on engraftment and transdifferentiation in old infarcted rats

Our results showed that hADSC administration in either young or old rats is effective in eliminating inflammation activity at day 3 and cardiac fibrosis at day 28 after MI. However, the extent of the improvement is greater in young compared with old. Cell therapy has shown promise to ameliorate heart failure following MI. However, experimental results of cell therapy for MI have not been as promising in older patients as in younger patients.^30^ We have quantitatively determined that recipient age has a dramatic influence on the seeding efficiency of hADSCs in the heart of old rats which is only 40% that measured in young rats (Figure 1A). Our data suggest that hADSC engraftment and differentiation change as a function of age‐related mechanisms. Environmental influences on stem cell function were shown in a heterochronic parabiosis experiment, in which the circulatory system of a young mouse was directly connected to that of an old mouse.^31^ The results demonstrated that exposing ageing stem cells to systemic factors derived from the young mouse increased their proliferative capacity and ability to contribute to muscle regeneration after injury.^31^ Our results were consistent with the findings of Suzuki et al,^13^ showing that the survival of the grafted cells might be reduced by the acute inflammatory response mediated by IL‐1β. A previous study reported that skeletal myoblasts expressing an IL‐1 inhibitor had a more than sixfold better survival rate after transplantation into infarcted myocardium than control cells at 3 weeks, and that this was associated with reduced fibrosis.^15^ Our results were consistent with previous studies, showing that co‐injection of skeletal myoblasts with superoxide dismutase into mice after MI reduced IL‐1β levels and improved cell survival.^32^ Targeting IL‐1 as a molecule modulating the pathological heart remodelling could therefore have an important clinical relevance.

hADSCs control excessive inflammatory responses by modulating NLRP3 inflammasomes. Human mesenchymal stem cells (hMSCs) have been shown to negatively regulate NLRP3 inflammasome activation in mouse macrophages by attenuating ROS production in mitochondria.^33^ However, Oh et al^34^ showed that the attenuated ROS by hMSCs administration had no effect on NLRP3 and IL‐1β expression at mRNA levels in macrophages. The discrepancy could be explained by the differences between in vivo and ex vivo. Compared with ex vivo experiments, NLRP3 and IL‐1β are up‐regulated and activated early after MI in a variety of cell types in the heart, not only infiltrating macrophages, but also fibroblasts, endothelial cells and border zone cardiomyocytes in the in vivo studies.^8^, ^9^ There were different responses of NLRP3 and IL‐1β to attenuated ROS in the different cells. Thus, it is not surprising to know that attenuated ROS production by hADSCs can reduce the NLRP3 and IL‐1β expression at mRNA levels in the in vivo study.

### 4.3 Effect of host pre‐conditioning by BP on hADSC engraftment

Our results revealed that the ageing infarcted rats pre‐conditioned by BP showed further reduced inflammation activity at day 3 and cardiac fibrosis at day 28 after hADSC administration compared with without BP pre‐conditioning. The detrimental effects of inflammaging on implanted stem cells can be pharmacologically reversed. In the current study, hADSC delivery into BP‐pre‐treated old hosts decreased active IL‐1β by 34% compared with hADSCs alone (Figure 3C). IL‐1β has been shown to play a role in acute inflammation and graft death after direct intramuscular cell transplantation to the heart.^13^ Our results showed in experiment 2 that BP pre‐treatment before stem cell implantation provided less inflammation microenvironment as shown reduced ROS levels and less NLRP3 inflammasome activity. The permissive microenvironment was abolished after adding SIN‐1, indicating that ROS production triggered NLPR3 inflammasome activation Thus, BP administration can rejuvenate the ageing rat heart via a ROS/NLRP3‐dependent mechanism. Attenuated NLRP3 inflammasome activity either by hADSC transplantation or by BP pre‐conditioning hosts can attenuate cardiac fibrosis. Interestingly, though both treatments share the same pathways, concomitant treatment provided additional benefits on decreased IL‐1β activity. After a targeted change in the ROS/NLRP3 axis in old rat hearts by primed BP, the ageing hearts were protected against stem cell necrosis. Our results were consistent with a recent study, showing that pharmacologic inhibition of NF‐κB, an upstream molecule of NLRP3 inflammasome, can rejuvenate stem cells in ageing hosts.^35^ Providing the proper environment for stem cell survival and host tissue integration is crucial in myocardial repair strategies.

### 4.4 Clinical implications

Considering the high global rate of age‐associated heart disease along with the rapidly increasing elderly population, the question of whether cardioprotection by hADSCs is maintained in ageing cohorts is crucial. Yet, the vast majority of research fuelling our understanding of the mechanisms is conducted in young animals. An older age has been reported to be a powerful independent predictor of mortality and morbidity in patients after a MI.^35^ Since the identification of factors that play important roles in cardiovascular regeneration and repair and those that become dysregulated with age, a number of therapeutic strategies to restore cardioprotective pathways in an older host have been developed. Of these, strategies that hinder the molecular pathways that play roles in cardiac ageing have been reported to potentially be able to reverse cardiac dysfunction and possibly reverse or delay the onset of chronic cardiovascular conditions. Our study implicates an essential axis of ROS‐NLRP3‐IL‐1β pathway in cardiovascular diseases associated with ageing, which reveals a potentially novel option involving use of ROS inhibitors, which target priming and activation steps of NLRP3 inflammasomes. Second, host tissue incompetence is an important problem in regenerative medicine since pre‐clinical therapeutic studies usually involve young animals, whereas stem cell therapy usually involves older patients. The discrepancy of engraftment efficacy between clinical and basic researches may be explained at least in part by recipient ages. Third, modification of the host environment may be a translational approach to functionally improve the regenerative capacity. Several methods have been reported to improve the survival of implanted cells, including Akt or bcl‐2 gene transfection, depending on donor cell level.^36^, ^37^ However, such approaches may not be clinically applicable due to the difficulty in delivering genes to the myocardium. Therefore, interventions aimed at improving the quality of the host micro‐environment by administering BP to facilitate survival and biological behaviour of implanted cells may be effective and clinically applicable.

### 4.5 Study limitations

There are limitations in the translation of the results of this study to other species and to human physiology. First, extensive stem cell death upon transplantation has been reported even in the native microenvironment as many experiments prior to transplantation are conducted in young hosts. Further research that creates a microenvironment that mimics the environment of natural tissues such as atherosclerosis is necessary in order to identify the required survival signals. Second, an emerging important question in stem cell transplantation is the timing of transplantation. Previous studies have shown that this different efficacy of stem cell implantation may partly result from different timing as there is a time‐dependent inflammation course post‐MI. After 1 hour post‐MI, massive myocardial necrosis, leukocytes and mast cells rapidly infiltrate into the ischemic myocardium,^38^ which may do harm for the survival of the implanted cells. That is why we chose stem cell implantation 1 hour after MI, during which maximal benefits of anti‐inflammation effect can be observed. However, our results may not be applicable to different time periods. Third, whether the decrease in cardiac fibrosis was caused by dead cardiomyocyte replacement or through the direct effects of paracrine factors released from the transplanted stem cells on the extracellular matrix was unclear.

## 5 CONCLUSIONS

In the present investigation, we identify ageing‐associated NLRP3 inflammasome as the cause of stem cell implantation failure and provide mechanistic insights into its reversal. This study provides evidence that BP can rescue an important cardioprotective pathway, and this may lead to the development of new preventive and therapeutic strategies for age‐related cell therapy.

## CONFLICT OF INTEREST

The authors confirm that there are no conflicts of interest.

## AUTHORS’ CONTRIBUTIONS

TML and HJH designed and performed most of the experiments, analysed and interpreted the data, and wrote the manuscript. TWC, MHC, CHC, PCL and SZL assisted during the acquisition, analysis, and interpretation of data and revised the manuscript. CHC assisted with data analysis and revision of the manuscript. TML is responsible for the integrity of the work as a whole. All authors reviewed and approved the final version of the manuscript.

## Supporting information

Supinfo

## Data Availability

The data that support the findings of this study are available from the corresponding author upon reasonable request.
