# Supplementary material for: Host pre‐conditioning improves human adipose–derived stem cell transplantation in ageing rats after myocardial infarction: Role of NLRP3 inflammasome
Source: J Cell Mol Med. 2020 Oct 6;24(21):12272–84. doi: 10.1111/jcmm.15403 (PMC7686984; doi:10.1111/jcmm.15403)
Supplement: Supplementary file 1 — Supinfo [file JCMM-24-12272-s001.docx]

***Characterization of hADSC surface phenotype***

Trypsinized hADSCs were suspended in 100 μL phosphate buffered saline. The cells (1×10^5^ per sample) were treated at room temperature for 20 min with the following specific anti-human antibodies: anti-Isotype IgG1-PE, -CD19-PE, -CD34-PE, -CD73-PE, -CD105-PE, -Isotype IgG1-FITC, -CD45-FITC, -CD90-FITC, -Isotype IgG2a-PE, -CD14-PE, -HLA-DR-PE (BD Biosciences, San Jose, CA, USA). Mouse IgG was used as a negative control condition. Fluorescent labeling was analyzed with a flow cytometer (Accuri C6; BD Biosciences, San Jose, CA, USA). The cell quality has been approved by the authority in Taiwan. Besides, the hADSCs have been used in clinical trial for liver cirrhosis (Topic: Clinical Trial Study About Human Adipose-Derived Stem Cells in the Liver Cirrhosis; ClinicalTrials.gov Identifier**:** NCT02297867).

***Echocardiogram***

Echocardiography was performed at baseline before and then again at 3 and 28 days after surgery. Rats were lightly anesthetized with intraperitoneal injection of Zoletil (20 mg/kg body weight) and xylazine (9 mg/kg). Echocardiographic measurements were done using the GE Healthcare Vivid 7 Ultra-sound System (Milwaukee, WI) equipped with a 14-MHz probe as previously described [1]. LV M-mode tracing was obtained using parasternal long-axis imaging, LV end-diastolic and end-systolic diameter dimensions were measured, and the fractional fraction (%) was calculated. Hemodynamics of the hearts were then quickly measured after systemic heparinization.

***Hemodynamics and infarct size measurements***

After echocardiography, the hemodynamics of the anesthetized rats were measured. Following the insertion of a polyethylene Millar catheter into the LV, five consecutive LV systolic and diastolic pressure cycles were measured via a transducer (Model SPR-407; Millar Instruments, Houston, TX, USA) as described previously [1]. The maximum rates of LV pressure rise (+dP/d*t*) and fall (−dP/d*t*) were measured, and then the atria and the right ventricle were removed. The LV was subsequently rinsed in cold physiological saline, weighed, and immediately frozen in liquid nitrogen after a coronal section of the LV had been obtained to estimate the infarct size. The sections were stained with trichrome and hematoxylin and eosin and, and the size of the infarct was determined as previously described [2]. To focus on clinically relevant conditions, only rats with large infarcts (>30%) were used for the analysis.

***Quantitative PCR (qPCR) of human Alu, p65 NF-κB, NLRP3, and IL-1β***

qPCR was performed from samples obtained from the border zone with the TaqMan system (Prism 7700 Sequence Detection System, PE Biosystems) at day 3 as previously described [1]. Primers sequences were the following:

human *Alu* sense 5'-CATGGTGAAACCCCGTCTCTA-3', antisense 5'-GCCTCAGCCTCCCGAGTAG-3';

p65 *NF-κB* sense 5'-CCTAGCTTTCTCTGAACTGCAAA-3', antisense 5'-GGGTCAGAGGCCAATAGAGA-3';

*NLRP3* sense 5'-CTGCATGCCGTATCTGGTTG-3’, antisense 5'-GCTGAGCAAGCTAAAGGCTTC-3’;

*IL-1β* sense 5'-ATGGCAACTGTCCCTGAACTCAACT-3', antisense 5'-CAGGACAGGTATAGATTCAACCCCTT-3';

*cyclophilin* sense 5’-ATGGTCAACCCCACCGTGTTCTTCG-3’, antisense 5’-CGTGTGAAGTCACCACCCTGACACA-3’.

Standard curves were plotted using threshold cycle against log template quantity. Following initial denaturation, the following 45 cycles of amplification were performed: 10 s at 95°C, 5 s at 60°C, and 10 s at 72°C. Fold change was normalized against *cyclophilin*, as the housekeeping gene.

***Immunohistochemical analysis of human mitochondria and sarcomeric 𝛼-actinin***

Immunohistochemistry staining for human mitochondria antibody, sarcomeric 𝛼-actinin antibody and 4′,6-diamidino-2-phenylindole dihydrochloride (DAPI) was used to identify and transdifferentiate the transplanted cells at the border zone on day 28 according to the manufacturer's protocol. The specificity of the antibodies in rats had been tested. Directly conjugated antibodies with identical isotopes were used as negative controls. The average of 10 random scans per section was used in the analysis. Staining quantification was calculated as the percentage of positively stained area/total area (%) at 400x magnification.

***Morphometry of cardiac fibrosis***

Picrosirius staining (Sirius Red F3BA; Pfaltz & Bauer, Stamford, CT), a specific method to detect collagen, and aniline blue staining were used to stain 5-µm-thick paraffin-embedded coronal sections of the remote zone (>2 mm within the infarct) at day 28 after MI. Quantitative morphometry of the picrosirius-stained sections was used to determine the interstitial collagen fraction using an automated image analyzer (Image Pro Plus, CA). These values were measured by at least two investigators who were blinded to which group the specimens belonged. Ten randomly selected fields were used to qualitatively estimate the density of labeled areas at 400× magnification. Values were expressed as labeled area/total area.

**References.**

1. Lee TM, Harn HJ, Chiou TW, Chuang MH, Chen CH, Lin PC, Lin SZ (2017) [Targeting the pathway of GSK-3β/nerve growth factor to attenuate post-infarction arrhythmias by preconditioned adipose-derived stem cells.](https://www.ncbi.nlm.nih.gov/pubmed/28130118) J Mol Cell Cardiol 104:17-30.

2. Pfeffer MA, Braunwald E. Ventricular remodeling after myocardial infarction. Circulation 1990;81: 161-172.
